# Mechanical stretching changes crosslinking and glycation levels in the collagen of mouse tail tendon

**DOI:** 10.1074/jbc.RA119.012067

**Published:** 2020-06-16

**Authors:** Melanie Stammers, Izabella S. Niewczas, Anne Segonds-Pichon, Jonathan Clark

**Affiliations:** Babraham Institute, Cambridge, United Kingdom

**Keywords:** Tendon, collagen, crosslinks, glycation, physical strain, chemistry, mechanical stress, imine bond, connective tissue, protein crosslinking, stress

## Abstract

Collagen I is a major tendon protein whose polypeptide chains are linked by covalent crosslinks. It is unknown how the crosslinking contributes to the mechanical properties of tendon or whether crosslinking changes in response to stretching or relaxation. Since their discovery, imine bonds within collagen have been recognized as being important in both crosslink formation and collagen structure. They are often described as acidic or thermally labile, but no evidence is available from direct measurements of crosslink levels whether these bonds contribute to the mechanical properties of collagen. Here, we used MS to analyze these imine bonds after reduction with sodium borohydride while under tension and found that their levels are altered in stretched tendon. We studied the changes in crosslink bonding in tail tendon from 11-week-old C57Bl/6 mice at 4% physical strain, at 10% strain, and at breaking point. The crosslinks hydroxy-lysino-norleucine (HLNL), dihydroxy-lysino-norleucine (DHLNL), and lysino-norleucine (LNL) in-creased or decreased depending on the specific crosslink and amount of mechanical strain. We also noted a decrease in glycated lysine residues in collagen, indicating that the imine formed between circulating glucose and lysine is also stress labile. We also carried out mechanical testing, including cyclic testing at 4% strain, stress relaxation tests, and stress-strain profiles taken at breaking point, both with and without sodium borohydride reduction. The results from both the MS studies and mechanical testing provide insights into the chemical changes during tendon stretching and directly link these chemical changes to functional collagen properties.

In the field of collagen research there remains a lack of understanding of how the different crosslinks found in collagen contribute to function. The classes of collagen crosslinking are described as immature, mature and advanced glycation end products (AGEs) (1). The immature crosslinks are formed through the action of lysyl oxidase on lysine and hydroxylysine residues to generate aldehydes which then react with nearby lysine or hydroxylysine side chain amines to form imine crosslinks (2). These can then react further with another oxidized lysine to form mature crosslinks (the pyridinolines) (2). AGE crosslinks are formed through the spontaneous reaction of circulating sugars with lysine, hydroxylysine, and arginine through chemical steps which have yet to be identified (1).

Although both the mature and AGE crosslinks are chemically stable, the situation for the immature imine crosslinks is uncertain. They are often described as acid labile or hydrothermally labile, but whether they hydrolyze under stress is less clear. It has been concluded previously through physical testing that the imines in tendon must be hydrolysable under stress (3, 4), although the importance of this in the mechanical properties displayed by tendon or whether they are really stress labile has subsequently been questioned (5, 6). We aim to shed light on the status and reactivity of these bonds in the work presented here through the direct measurement of these crosslinks at different strains in 11-week C57Bl/6 mouse tail tendon.

Increases in AGE crosslinks and mature crosslinks with age are generally considered to cause increases in tendon stiffness (1). We have previously shown that the glycation of lysine residues within the collagen structure are also likely to contribute to collagen stiffening (7). Glycated lysine is not considered a covalent crosslink. The chemistry of glycated lysine is similar to that of the immature crosslinks in originating from the chemical reaction of a sugar aldehyde with an amine to form an imine. In the light of this similarity, we included analysis of glycated lysine in this study.

To understand the role of reversibility of the imine bonds in the normal functioning of tendon, we use sodium borohydride reduction to convert the imines into bonds which cannot be broken. This preserves the number of crosslinks and glycated lysine residues as well as their position in the collagen structure. We then compare the results of mechanical testing of reduced samples where the chemical bonds cannot break under stress with the results from unreduced tendon where the crosslinks and glycation could break under stress to look for differences in the stress-strain (stress is force/cross-sectional area; strain is extension/initial length) profiles.

For clarity in this paper we describe the immature bonds in terms of the reduced products that we actually measure by HPLC MS, dihydroxy-lysino-norleucine (DHLNL), hydroxy-lysino-norleucine (HLNL), and lysino-norleucine (LNL) (structures shown in Fig. S1).

**Figure 1.**
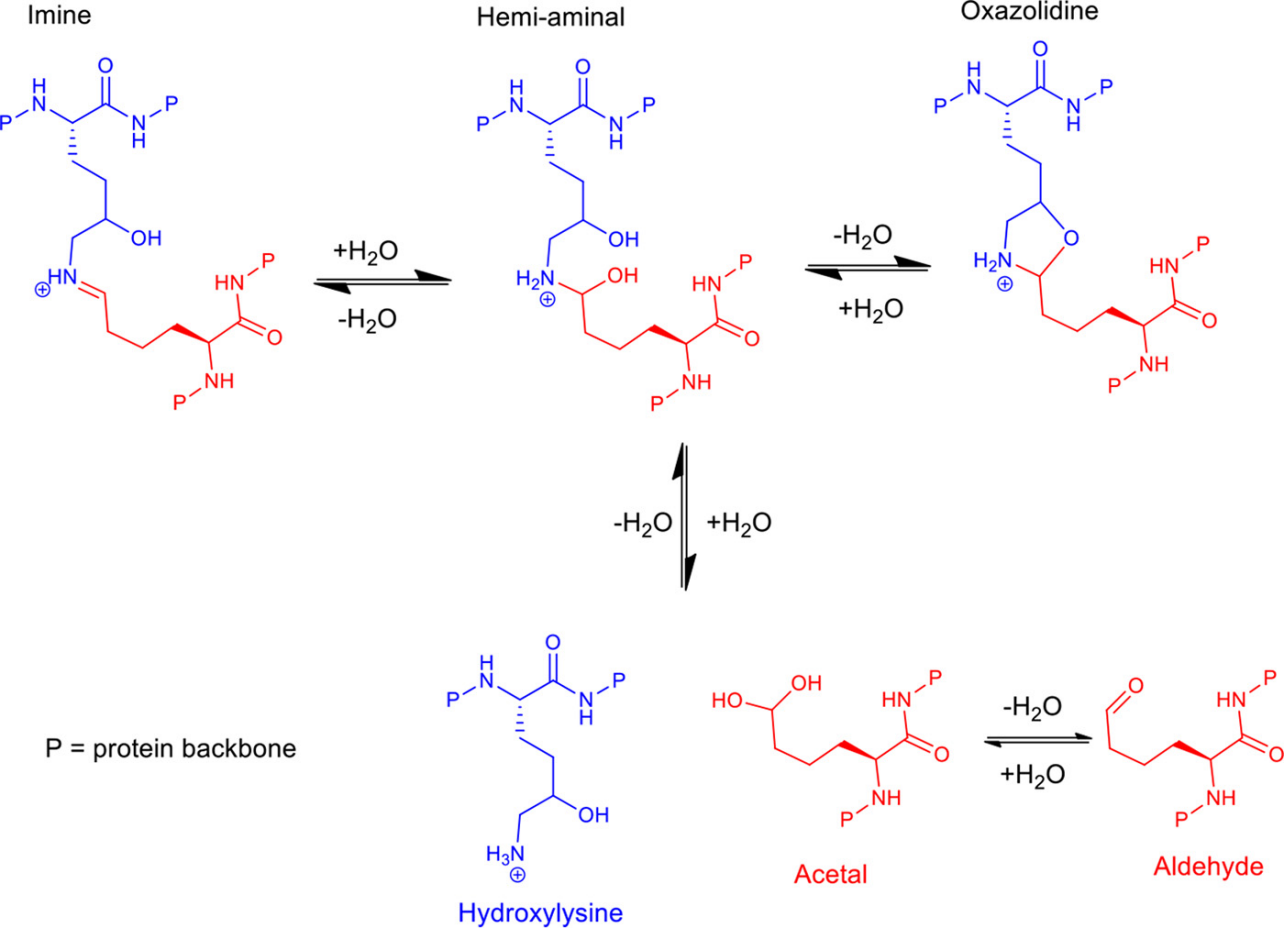
**Scheme showing possible structures in the equilibrium between aldehyde (*red*) and hydroxylysine (*blue*) residues in collagen.** The imine, hemi-aminal, and oxazolidine are possible crosslinks that may exist in equilibrium with each other. None of these crosslinks have been observed in native collagen to date, possibly because of their low abundance and that current NMR spectrometers do not yet have the required sensitivity.

## Results

### Changes in tendon stress-strain profile by chemical treatment

Imines in an aqueous system are expected to be in an equilibrium, as shown in Fig. 1. The addition of sodium borohydride will reduce the imine and the aldehyde in this equilibrium to a secondary amine and alcohol, respectively, irreversibly fixing the crosslink equilibrium at that point. These reductions are the only reactions that will occur in tendon under the conditions used here. If collagen chemistry is changing under tension, then adding sodium borohydride to tendons should change the stress-strain profile seen because the crosslinking can no longer change. While the reduction of an imine to a secondary amine might in itself cause a change in the profile because of increased rotation about a single bond verses a double bond, this might be expected to decrease tendon stiffness. Increases in tendon stiffness and increases in permanent deformation on stretching would be signs of change corresponding to the conversion of a reversible bond into an irreversible one.

### Demonstration of change in tendon stress-strain profile when cycling to 4% strain with and without reduction

**Figure 2.**
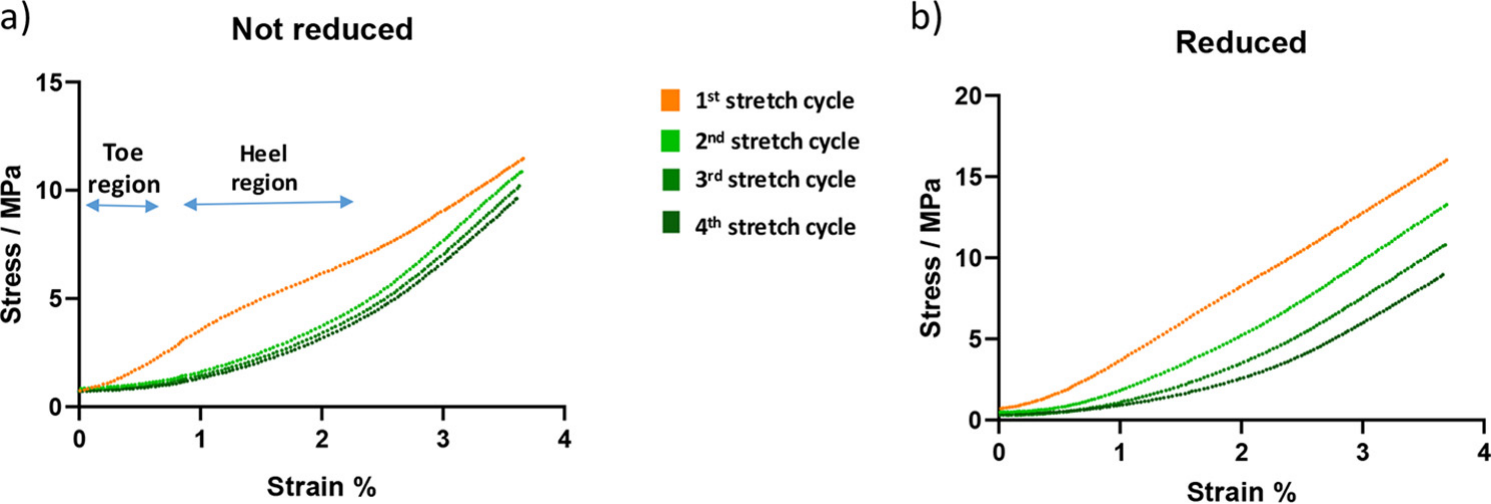
*a*, typical stress-strain profile of unreduced tendon; this was repeated 27 times. Further examples where no toe–heel region are observed on stretching of unconditioned tendon can be seen in Fig. 4*b* (*orange traces*). *b*, typical stress-strain profile of reduced tendon; this was repeated eight times. Each plot shows four stretch cycles taken to a strain of 4%. The tendon was preloaded to 0.01 N at the start of testing. Tendon in both examples taken from 11-week-old C57BL/6 mice. The *orange trace* is the first cycle in each plot; subsequent cycles shown in *green*.

Fresh tendon was stretched to 4% strain and back to zero in a tensile stress stage through four cycles with 15-min rests between each cycle. The process was then repeated with tendon which had been reduced with sodium borohydride for 1 h prior to testing. The unreduced tendon showed a large change in stress-strain profile between the first cycle and second cycle but then subsequent cycles showed very similar stress-strain plots, Fig. 2*a*. We found that this difference between the first test cycle and subsequent cycles is consistently observed. The toe and heel regions seen in cycles 2 onward are commonly referred to in the literature (8); however, in our experience they are not seen clearly unless a pretest stretch is carried out first, a procedure that is commonly described to “condition” tendon before experimentation.

Reduced tendon behaves quite differently on repeated testing to 4% strain (Fig. 2*b*). Repeated cycles show a continual plastic deformation between each testing cycle (a plastic deformation is a nonreversible change). This is shown by the recorded stress starting to increase at higher strains with each test cycle, indicating that the tendon length has increased slightly on each cycle because of deformation. The reduced tendon does not recover on each cycle repeat, whereas the tendon that had not been reduced shows an ability to largely recover from the second cycle onward.

### Stress relaxation tests show continued change after first cycle

**Figure 3.**
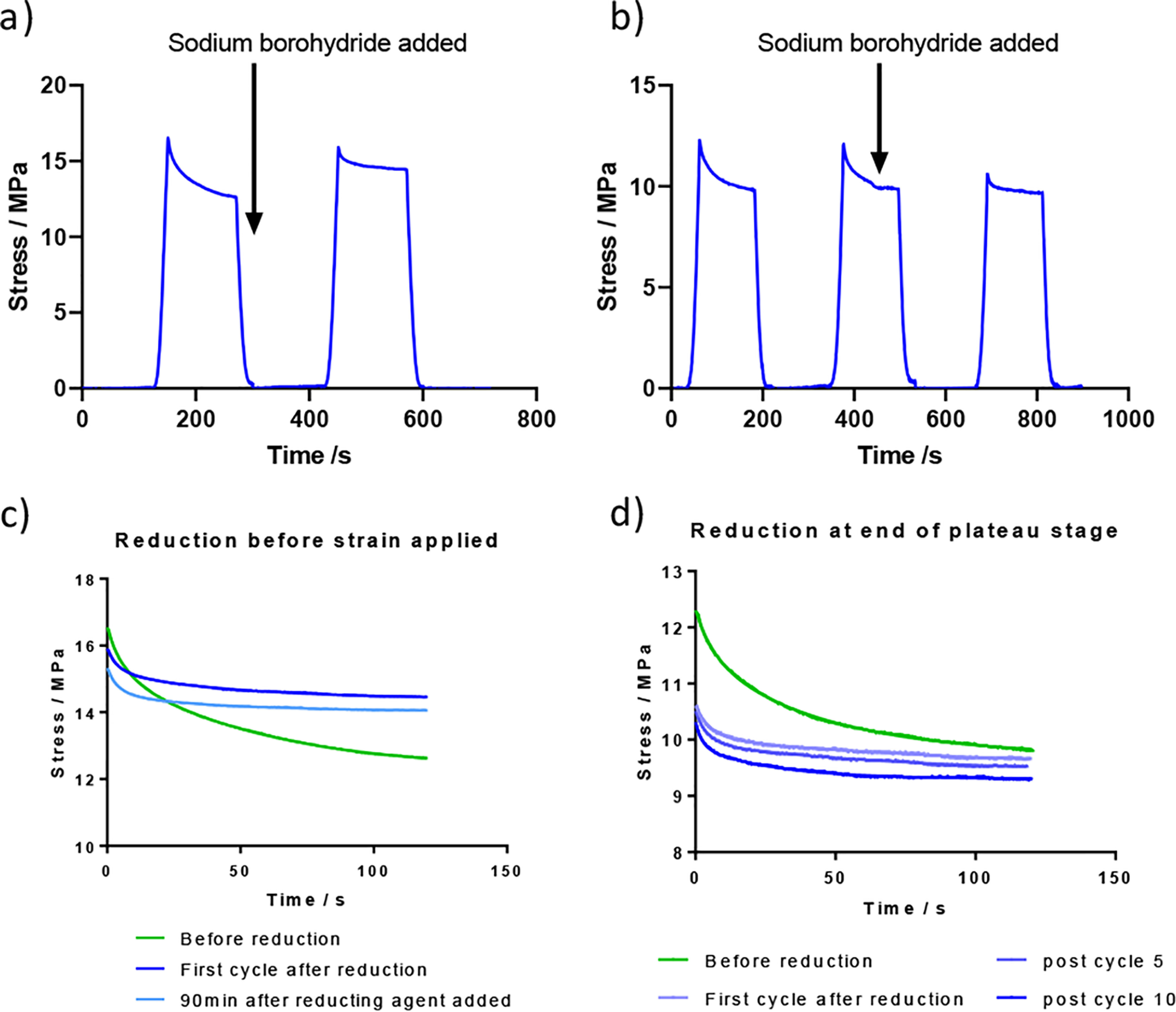
**The stress relaxation testing from tendon fibers from 11-week-old C57BL/6 mice prior to, and after exposure to, a reducing agent (sodium borohydride) that fixes the reversible bonds.** This was repeated six times. *a* and *b*, the reducing agent addition is shown. *c* and *d*, overlap plots of the relaxation curves are shown in the lower traces. Tendon was conditioned by four prestretching cycles before use.

Tendons were preconditioned by stretching to 3% strain four times before experimentation. The tendon was then repeatedly extended to 3% strain and held for 120 s while the decrease in stress (stress relaxation) was monitored before returning to the initial length. Fig. 3, *a* and *c*, demonstrates the effect when sodium borohydride was added during the rest period after the first test cycle. It can be seen that the stress profile in the cycle after treatment is different to that before treatment. The stress drops during the 120 s period (because of structural relaxation processes) in both cases, but after reduction the stress does not drop as far during this period as that seen before treatment, although the initial stress on extension is similar. This shows that fixing the crosslinks by reduction so that they cannot break decreases the ability of tendon to relax when stretched.

In the experiment shown in Fig. 3, *b* and *d*, the sodium borohydride was added 60 s into the 120-s stress relaxation period. In this case it can again be seen that the profile in subsequent cycles is changed by the reduction step. The initial stress achieved after reduction is lower than that seen before reduction whereas the stress measured in the plateau phase is similar. This shows that the network of crosslinks has been fixed while the tendon is in an extended state and cannot recover the original structure.

These two experiments show that the crosslink network is changing and can be chemically fixed by reduction at different points in the stress-relaxation profile. This implies that chemical change is occurring even after preconditioning stretches.

### The plastic phase is lost on reduction

**Figure 4.**
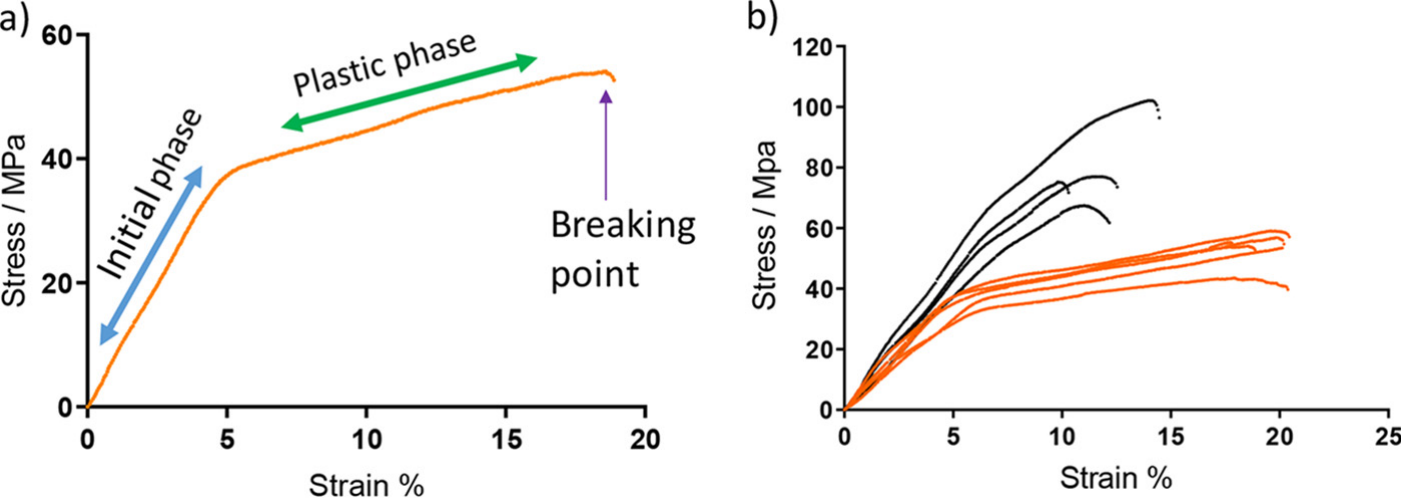
*a*, a typical stress-strain profile for 11-week-old C57BL/6 mouse tail tendon. *b*, profile showing overlay of reduced (*black*) and unreduced (*orange*) 11-week-old C57BL/6 tendon showing the loss of the plastic region on reduction. Tendon used was unconditioned.

Tendon, particularly from young animals, exhibits a plastic phase after a strain of 5% (Fig. 4*a*). Fig. 4*b* shows a comparison of the break test profiles of unreduced and reduced tendons (without preconditioning). A loss of the plastic phase in the profile of the reduced tendon when compared with unreduced tendon is seen. It is also clear that a higher final stress is reached in the reduced tendon. This shows that the plastic deformation phase is lost when the crosslinks cannot break and also that higher stresses can be achieved when the crosslinks cannot break.

### Crosslinking and glycation change under stress

To demonstrate that bonds break and form under stress, the change in crosslink levels at defined strains were measured. We chose to make measurements at 4% strain, 10% strain, and at breaking point (BP). These were chosen as representative points on a typical mouse tail tendon stress-strain profile as illustrated in Fig. 4*a*. The tendon was held at the defined strain for 10 min to maximize any chemical changes occurring.

**Figure 5.**
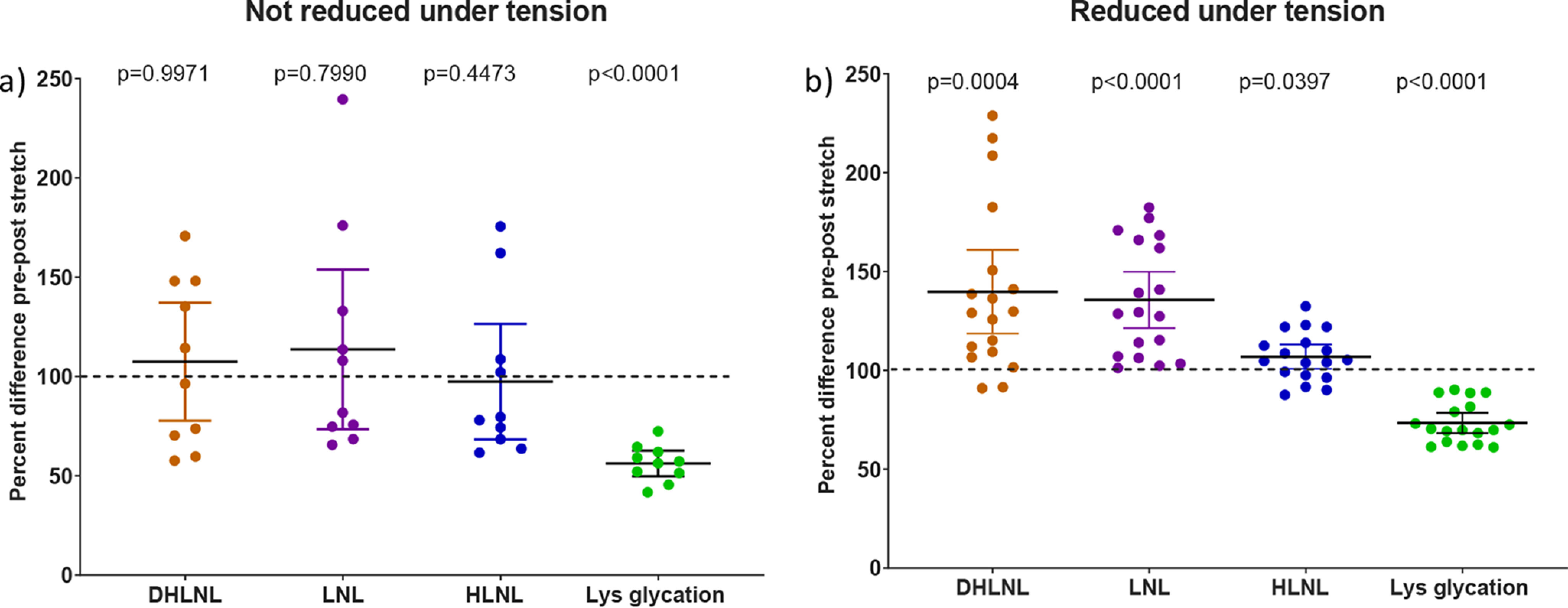
*a*, the change in analytes on stretching tail tendon fibers of C57BL/6 mice to 4% strain with reduction after removing from testing apparatus (mean ± 95% CI; *n* = 10 mice). Statistical analysis: two-sided ratio Student's paired *t* tests. *b*, the change in analytes on stretching tail tendon fibers of C57BL/6 mice to 4% strain with reduction before removing from testing apparatus (mean ± 95% CI; *n* = 18 mice). Statistical analysis: two-sided ratio Student's paired *t* tests. Tendon used was unconditioned. The structures of LNL, HLNL, and DHLNL are shown in Fig. S1. Lys-glycation (the addition of a hexose to the side chain NH_2_ of lysine).

The initial experiment is shown in Fig. 5*a* where tendon was taken to a 4% strain, cut out of the equipment, and then reduced with sodium borohydride. In each case a sample of tendon was removed before stretching to act as the control with which to compare the crosslink level in the stretched tendon. This experiment did not show any convincing change in the mean for immature crosslinks, although the glycation of lysine was seen to decrease.

**Figure 6.**
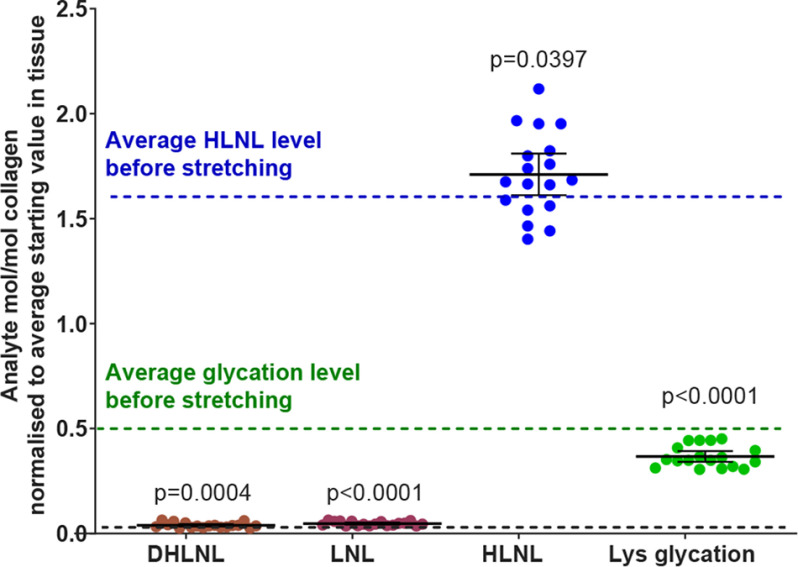
**Graph shows the change in analytes on stretching tail tendon fibers of C57BL/6 mice to 4% strain normalized to the average mol/mol collagen value before stretching (mean ± 95% CI; *n* = 18 mice).** Statistical analysis: two-sided ratio Student's paired *t* tests. This plot highlights the relative abundance of each analyte.

The spread of data points around the mean was greater than we would have expected, which led us to question whether the rate of change was fast and whether we needed to trap the reaction in the stretched state. Further experiments were carried out where the tendon was reduced *in situ* while still under strain to fix the tendon chemistry present at that point. The previous experiment was repeated with this modification to the reduction procedure and the result can be seen in Fig. 5*b*. Clear changes can now be seen in all the analytes shown. The data in Fig. 5 are displayed as a percentage change; however, the DHLNL and LNL are found in tendon at very low levels. Fig. 6 shows the same data as in Fig. 5*b* but normalized to the average mol/mol collagen level for each analyte on the same scale so that the relative contributions of each crosslink type can be assessed. This figure is to emphasize that a seemingly large and significant percentage change in analyte level can represent quite a small absolute change relative to other analytes.

**Figure 7.**
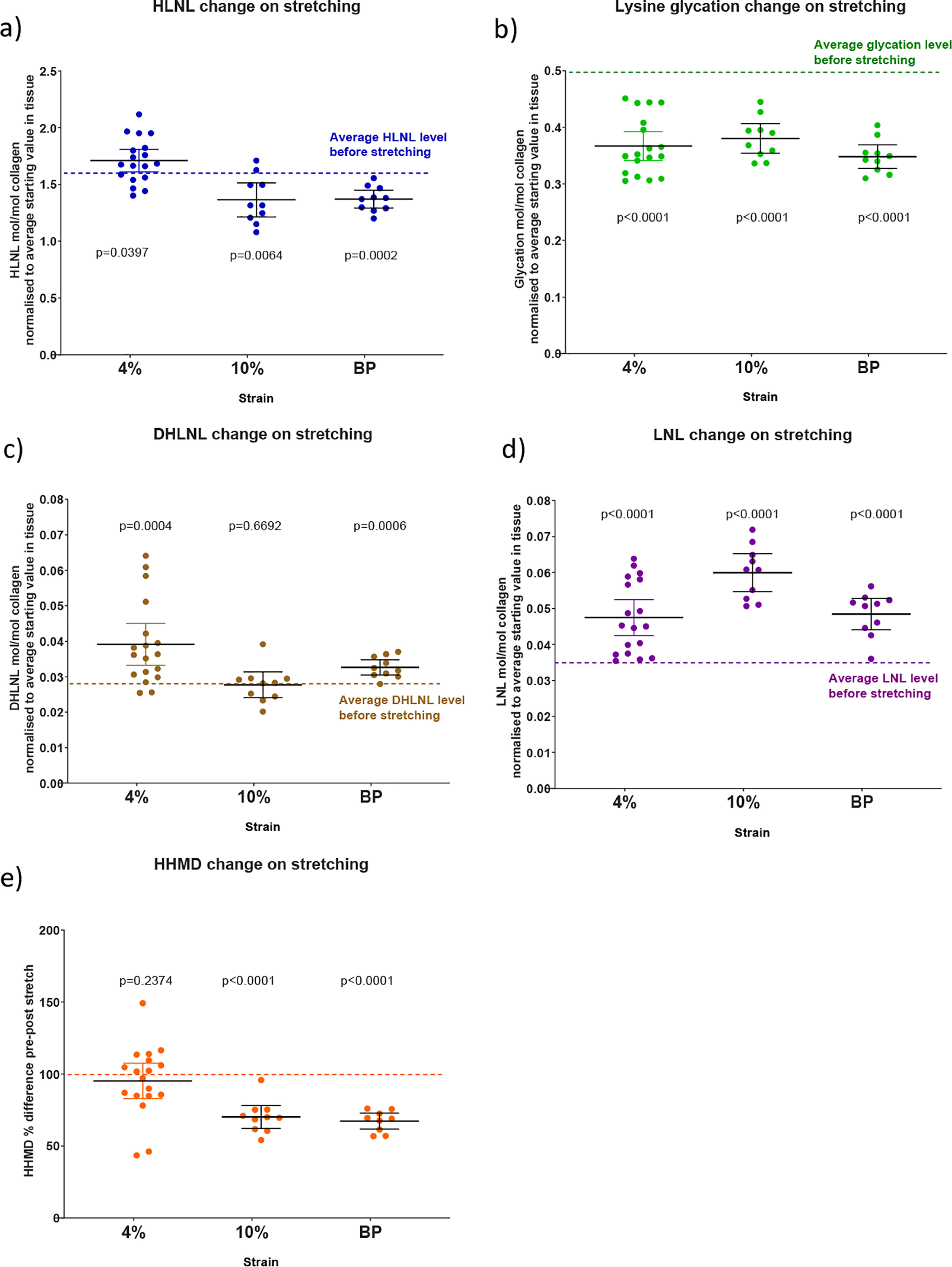
**Graphs to show the change in analytes on stretching tail tendon fibers of C57BL/6 mice (mean ± 95% CI; *n* = 10 except 3a where *n* = 18 mice).** *a*–*d*, values normalized to the average mol/mol collagen value before stretching. Statistical analysis: two-sided ratio Student's paired *t* tests.

Fig. 7, *a*–*e*, summarizes the data from the different stretching experiments grouped by analyte with the average starting level found in unstretched tendon shown. Each data point represents tendon from different animals. At 4% strain HLNL levels increase, although this increase only just reaches statistical significance. At 10% strain and BP, the HLNL levels show a clear decrease. Although the increases in DHLNL at 4% and at BP are small, they occur consistently. This is also true for LNL at all test strains. Lysine glycation drops at 4% strain and does not appear to decrease further with increasing strain.

Fig. 7*e* shows the result for the crosslink histidine-hydroxymerodesmosine (HHMD), which is formed through the reaction of two aldehydes, one hydroxylysine and a histidine. Although there is debate (9–11) about what HHMD actually represents in native collagen, it does reflect a reversible chemical change occurring within the tissue and is a structure that also contains an imine. A standard for this compound, which would allow quantification by MS, is not available and so the data have been expressed as a percentage change. Levels of HHMD have been reported to be as high as 0.89 mol/mol collagen by others (12) determined using tritium labeling.

The results described above collectively led us to a hypothesis that there might be free aldehyde derived from lysine oxidation (allysine) in resting tendon and that the levels of allysine increase on stretching. To explore this idea, a sample of tendon was taken to 10% strain, reduced under tension, and compared with the unstretched control. The level of reduced allysine in this sample before stretching was found to be 89 ng/mg tendon and after stretching 235 ng/mg tendon (dry delipidated tendon weights). The levels of the aldehyde derived from hydroxylysine were too low to be detected. (These results are summarized further in Fig. S2.)

## Discussion

Until the work described here, there was no proof that crosslinking within collagen can change in response to mechanical strain. We have demonstrated that crosslinking and glycation within tendon changes under strain. Both increases and decreases in the mean number of individual crosslinks were seen, which shows that both bond formation and bond breaking were occurring.

The cyclic stress-strain profiles in Fig. 2*a* show that the first testing cycle is quite different to subsequent ones. The loss of glycation is a permanent change which occurs during this stretch phase, Fig. 7*b*. In the light of this, it would seem plausible that the loss of glycation contributes to the initial drop seen between the first and second cycles and that glycation has a role in the stiffening of tendon during the early stretching phase.

In Fig. 2, *a* and *b*, it can be seen that reduction has a large impact on the profiles when tendon is stretched to 4% strain. The untreated tendon, after the first cycle, shows a remarkable reproducibility whereas the reduced tendon shows a marked deformation on each subsequent stretch cycle. This difference indicates that the ability for bonding to change in the unreduced tendon allows an adaptive change that largely prevents through stress relaxation (or mitigates through bond re-organization) the plastic deformation seen in reduced tendon.

The stress-relaxation studies show that adaptive changes occur under tension to allow relaxation and recovery processes to occur. When tendon is reduced the adaptive changes are stopped and the tendon is chemically fixed with the bonding network at the point of reduction. The only known chemistry that is fixed in collagen using sodium borohydride is that around the imine bond equilibrium illustrated in Fig. 1, indicating that it is this reversible chemistry that is responsible for the adaptive change.

Fig. 5*b* shows that a 4% strain can actually lead to an increase in the number of reversible crosslinks present in tendon under tension. This suggests a more ordered structure where crosslink-forming residues have been brought into proximity with each other, allowing crosslink formation to occur. This is in keeping with synchrotron X-ray scattering evidence, where it was suggested that strain created a more ordered structure and that the relaxation process was entropically driven (13).

These imine bond breaking and formation reactions are relatively fast, as shown by the experiment carried out by reduction of tendon in the relaxed state after stretching in Fig. 5*a*; however, the reaction rates in the equilibria are unknown. It is possible that the rate of bond hydrolysis is limiting, which may explain why higher stresses and an increased stiffness can be observed with faster rates of extension (an example is shown in Fig. S3).

In the plastic phase, the collagen fibers are believed to slip against each other. This was described previously by others in studies based on X-ray diffraction studies (14, 15). During this phase we find that there is a net decrease in HLNL bonds, and it would seem possible that this could be to allow an extension process to occur, for example in growth, or to reduce stress when over extension occurs. When the reversible bonds are fixed by reduction, the plastic phase can no longer occur and is lost from the profile, as seen in Fig. 4*b*. It can also be seen that higher stresses are then achieved because adaption to the stress cannot occur through bond breaking.

One consistent feature seen in the 4% strain experiments is an increase in DHLNL of between 0.006 and 0.01 mol/mol collagen when compared with unstretched tendon. This might seem small, but a 0.6 to 1% increase in crosslinking on stretching might have an important functional role, particularly if combined with the changes seen in LNL and HLNL. The result requires a pool of the aldehyde derived from hydroxylysine to be present. To prove this by detection of this aldehyde in these experiments would require the development of more sensitive detection methods not currently available, but it is reasonable to think that it is present, otherwise the increase in DHLNL seen would not be possible.

The increases seen in LNL on stretching at all strains tested may be a result of increased levels of aldehyde present in the tissue when stretched and the statistical chance of crosslink reforming with a lysine rather than a hydroxylysine. This increase in LNL could also be linked to structural changes involved in stretching during which the proximity of lysine or hydroxylysine to aldehyde changes.

The data shown in Figs. 2*a* and 7*b* indicate that tendon is stiffer when lysine residues in the collagen structure are glycated. A mechanism by which stiffening from glycation occurs is most likely through the interactions of the sugar component of the glycated lysine residues with adjacent collagen molecules, for example through hydrogen bonding of the sugar hydroxyls with nearby groups. If this did not occur then there would be no reason for the glycated lysine to hydrolyze when the tendon is stretched. There has to be a mechanism by which the transfer of strain can take place to lengthen the sugar-lysine imine bond, thereby lowering the activation energy of the bond toward hydrolysis. This transfer of strain requires at least two fixed points either side of the imine bond. The effect of glycated lysine appears to be important in intrafibrillar interactions rather than interfibrillar interactions because hydrolysis occurs during the initial 4% stretch phase with no further loss in the plastic phase when interfibrillar interactions become important.

**Figure 8.**
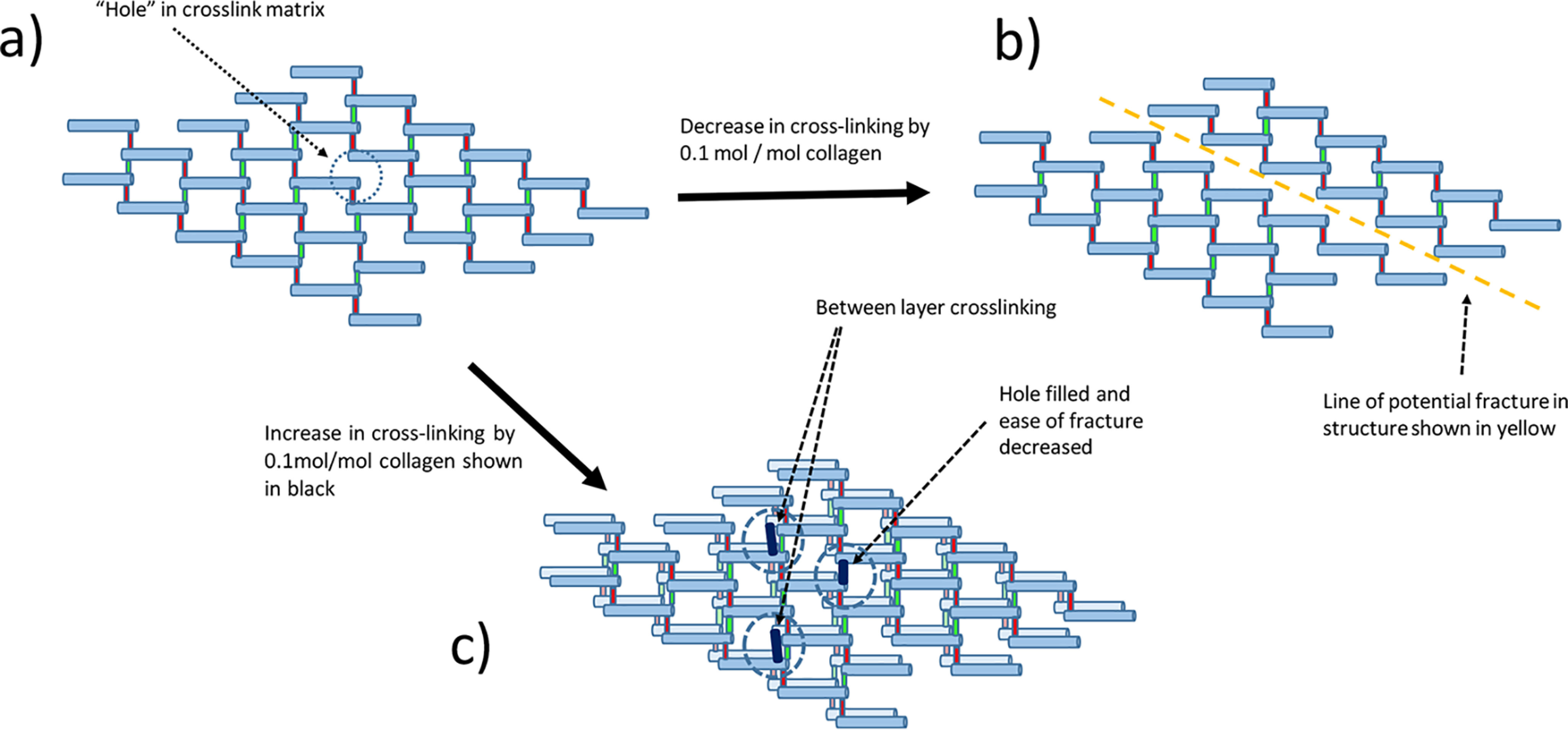
**A schematic model of collagen to illustrate the potential impact of a 0.1 mol/mol collagen change in crosslinking.** This model shows a typical initial level of 1.5 mol crosslinking/mol collagen in each layer. 30 collagen molecules per layer shown in *blue* with 30 crosslinks shown in *red* and assigned as unchanging with 15 crosslinks shown in *green* and assigned to be sites of bond formation and breakage. Five of the *red* cross-links which would be found at the terminus of the collagen chains and create a link to the next block of 30 collagen molecules are not shown. In this model, a decrease or increase of 0.1 mol/mol is equivalent to a change of three bonds per 30 collagen molecules. *a*, the top left model illustrates that at this level of crosslinking there are holes in the structure, *i.e.* the level of crosslinking is not sufficient to completely crosslink in a 2D plane. *b*, the top right model shows that when there is a decrease of 3 crosslinks in the model there is the potential for a fracture line to develop through the polymer. *c*, the bottom structure illustrates that when there is an increase of 3 crosslinks, the hole in this layer can fill and crosslinking then has to occur between layers, which would clearly make the overall structure much stronger.

The largest crosslink mol/mol collagen change is that seen for HLNL, ranging from an average increase of 0.1 mol/mol collagen under 4% strain conditions, to a decrease of 0.2 mol/mol collagen under 10% strain conditions. A schematic model is presented in Fig. 8 that shows five parallel crosslinked strands of six collagen molecules, each strand overlapping in a staggered formation. For clarity it is presented as a planar 2D structure, initially with a typical crosslink density of 45 crosslinks to 30 molecules of collagen (1.5 mol/mol collagen). It can be seen in Fig. 8*a* that at this level of crosslinking there is a crosslink gap in the matrix. Fig. 8*b* shows the potential impact of a decrease in crosslinking of 0.1 mol/mol collagen (3 crosslinks). It can be seen that this could greatly weaken the structure creating a fracture plane indicated by the *yellow line*. Fig. 8*c* shows that an increase of three bonds could clearly strengthen the structure by filling the crosslink gap and potentially forming crosslinks with adjacent collagen planes. Although the reality of the native collagen structure is far more complex and subtle than this 2D model, it illustrates the impact that changes on the scale that we have observed could have.

The evidence presented here shows that tendon collagen undergoes chemical changes in crosslinking and glycation under strain that are important for correct function. By using reduction with sodium borohydride which fixes the crosslinks, it was demonstrated that the reversibility of crosslink and glycation chemistry is essential for the proper mechanical properties of tendon. The picture that has emerged is one of highly dynamic crosslinks, breaking and re-forming with every stretch. Changes in the levels of crosslinking and glycation would be expected to impact tendon function, with implications for aging, disease, and sport sciences.

## Experimental procedures

### Animal procedures

#### 

##### Ethics

Animal experiments were performed according to the UK Animals (Scientific Procedures) Act 1986, license PPL 70/8303 and approved by the Babraham Institute Animal Welfare and Ethics Review Body.

### Sample analysis

#### 

##### Tissue processing

After removal of the skin from isolated tails, tendon was drawn out of the tail under PBS pH 7.4 by grasping the tip and the base of the tail with forceps. Twisting the forceps holding the smaller vertebrae at the tail tip caused the ligaments holding the vertebral bones together to break, allowing the vertebral bones with attached tendons to be gently pulled out. For larger vertebral joints they were first weakened by inserting the tip of a scalpel between the joints taking care not to cut the tendons. Tendon was isolated sequentially one vertebral bone at a time, working up the tail from the tip to the base. Tendon fibers were detached from the tail vertebrae with a scalpel.

##### Sample analysis

Acid hydrolysis and sample analysis by HPLC-MS was carried out as described previously (6).

##### Allysine analysis

For this analysis, sodium borodeuteride was used because the background noise and isobaric background was much lower with the increase in mass. Tail tendon (wet weight 10 to 20 mg) was prepared and reduced as for the other experiments described here but with NaBD_4_ used as the reducing agent. Base hydrolysis was then carried out in 200 μl 2m NaOH at 100°C for 24 h. The sample was then allowed to cool and 600 μl of 1m HCl added and the sample made up to 8 ml with 50% acetonitrile in water. The sample was then split in half and 6-hydroxy-norleucine (0.5 eq based on dry weight) added to one half. Both samples were then loaded onto disposable 500 mg SCX ion exchange columns (Sigma, cat. no. 57018). Each column was then washed with 2 ml 50% acetonitrile, then the analytes eluted with a mixture of 1.5 ml 50 mm ammonium carbonate pH 7.5 added to 50% acetonitrile in water. The samples were then freeze dried, redissolved in 400 μl 50% acetonitrile, filtered through a 0.22-μm nylon filtration membrane and freeze dried again. The samples were then taken up in 30 μl 50% acetonitrile in water and 5 μl analyzed using the HLPC-MS method and parameters described previously (1). The increase in nondeuterated signal area in the standard sample was used to work out the amount of deuterated analog in the sample to which no standard had been added for each sample pair.

The samples were then expressed as ng/mg dry delipidated tendon and an approximate mol/mol collagen value calculated based on the dry delipidated tendon mass assuming 100% collagen content. The resulting mol/mol collagen values would therefore be expected to be slightly lower than the actual values.

### Physical testing

#### 

##### Stress-strain stage setup procedure

Physical testing was undertaken using a Microtest 200 N tensile stress stage (Deben UK Ltd) fitted with a 20 N load cell and a Petri-dish bath to allow immersion of the sample. With a distance of 14 mm between the jaws, individual processed fibers were clamped submerged in PBS pH 7.4 at room temperature, and preloaded to a force of 0.01 N. The diameter of each fiber was assessed along its length and the smallest dimension used to calculate stress.

##### Cyclic strain testing

After the tendon had been loaded to 0.01 N and the diameter and length measured, the distance between the jaws was decreased by 0.05 mm. At this point the crimp pattern could be seen. After a 15-min rest, the tendon was taken to a 4% strain at a rate of 0.5 mm/min, held for 0.1 s, and then relaxed back to the cycle initial strain. The force was recorded every 0.5 s during the cycle. After a rest of 15 min the cycle was repeated. Four cycles were carried out on each tendon tested. Where tendon was reduced first, this was done by dissolving 8 mg NaBH_4_ in 10 μl 1 mm sodium hydroxide which was then added to 1 ml of PBS. This 1 ml solution was then added to the tendon in 4 ml PBS and left for 1 h with gentle agitation every 15 min. The reduced tendon was then washed twice in fresh PBS before testing.

##### Stress relaxation testing

A single tendon fiber after preloading to 0.01 N, was preconditioned by holding at an extension of 3% for 60 s and then taken back to its original length to rest for 60 s, and repeated a further three times. During the stress relaxation experiment the fiber was repeatedly extended to 3% for 120 s and then back to its original length to rest for 120 s, recording the force every 0.5 s. NaBH_4_ (4 mg in 40 μl 1 mm NaOH added to 1 ml PBS) was added by exchanging with 1 ml of PBS from the bath (18 ml total) and mixed by pipetting.

##### Break test profile measurements

Tendon was prepared as described above and taken to breaking strain at a rate of 1 mm/min. The force was recorded every 0.5 s.

##### Extension crosslink measurements

Tail tendon was removed from male mice as described in the processing method, except the tendons were left attached to the tail vertebrae for ease of handling. With the jaws of the tensile stress stage set 18 mm apart, five to eight vertebral sections were mounted into the jaws, totaling 20-28 tendons. These were clamped in place across the tendon fibers removing the vertebral bones once clamped and submerged in PBS at room temperature. Excess was cut away and used as a control. At a rate of 0.2 mm/min the fibers were taken to breaking point, after which the tendon was excised from the jaws and both the control and the broken tendon were reduced as above in 200 μl PBS. This process was repeated twice for each mouse tail in order to produce enough material for analysis. The tendon was subjected to acid hydrolysis and analysis by MS as detailed above.

For analysis at 4 and 10% strain, tail tendon was collected from mice as described above and clamped into the jaws of the tensile stress stage, submerged in PBS at room temperature, with the jaws of the tensile stress stage ∼18 mm apart and at an initial load of 0.01 N. Control material was collected by cutting away excess not held in the jaws. The jaws were extended to the defined strain at a rate of 0.1 mm/min and held at that strain for 10 min. The samples were reduced *in situ* under strain by exchange of 1 ml of PBS from the bath with 1 ml PBS containing NaBH_4_ (4 mg in 40 μl 1 mm NaOH added to 1 ml PBS) for 5 min in the bath, then excised from the jaws. Reduction was allowed to continue for a further 2 h in a tube. Samples collected were subject to acid hydrolysis for analysis as described above.

### Statistical analysis

For all stretching experiments, ratio paired Student *t* tests were used. For the other experiments, one-way analyses of variance were used when more than two groups were compared followed by Tukey's multiple comparisons tests. Significance was defined as *p* < 0.05 and *p*-values are reported on figures. Data analysis was performed using GraphPad Prism 8.

## Data availability

All data are available from the corresponding author upon reasonable request.

## Supplementary Material

Supporting Information
