# Supplementary material for: Mechanical stretching changes crosslinking and glycation levels in the collagen of mouse tail tendon
Source: J Biol Chem. 2020 Jun 16;295(31):10572–80. doi: 10.1074/jbc.RA119.012067 (PMC7397108; doi:10.1074/jbc.RA119.012067)
Supplement: Supporting Information [file supp_295_31_10572__index.html]

Mechanical stretching changes cross-linking and glycation levels in the collagen of mouse tail tendon — Changes in collagen crosslinks and glycation under strain — Mechanical stretching changes crosslinking and glycation levels in the collagen of mouse tail tendon — Changes in collagen crosslinks and glycation under strain — Supporting Information 

# Mechanical stretching changes crosslinking and glycation levels in the collagen of mouse tail tendon

## Supporting Information

- Supporting Information for collagen crosslinking and glycation changes on stretching - Supplimentary figures
