# Supplementary material for: Mechanical stretching changes crosslinking and glycation levels in the collagen of mouse tail tendon
Source: J Biol Chem. 2020 Jun 16;295(31):10572–80. doi: 10.1074/jbc.RA119.012067 (PMC7397108; doi:10.1074/jbc.RA119.012067)
Supplement: Supporting Information [file supp_RA119.012067_157371_3_supp_544992_qbntw5.pdf]

Supplementary figures follow:

Figure S1, The structures of LNL, HLNL, and DHLNL. Also showing the relationship to lysine and hydroxylysine and the intermediate crosslink structures found in collagen

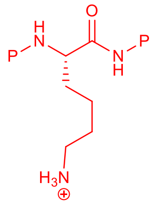

Lysine

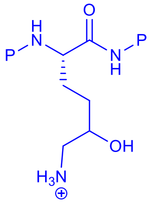

Hydroxylysine

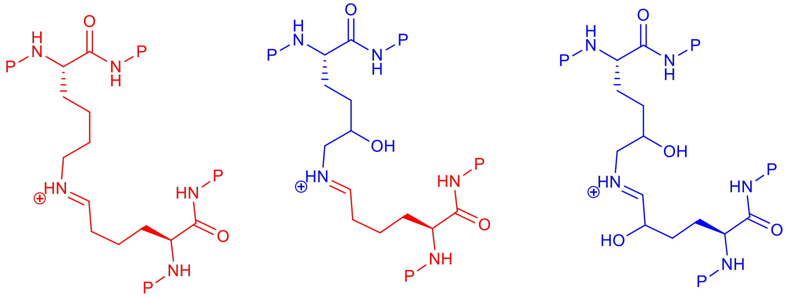

The imine form of crosslink that is reduced with sodium borohydride in collagen

P = protein backbone

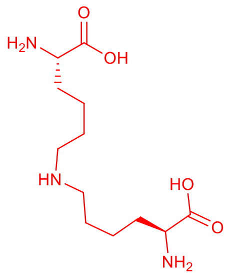

LNL

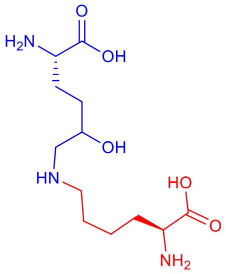

HLNL

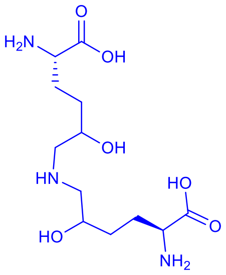

DHLNL

Analyte measured after sodium borohydride reduction

Figure S2 Allysine analysis

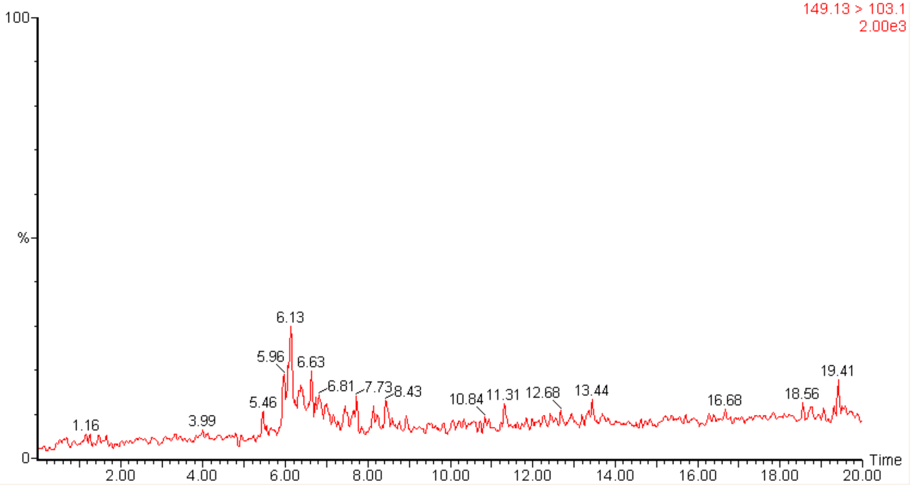

Above: m/z 149 trace showing data from unstretched tendon without a reduction step.

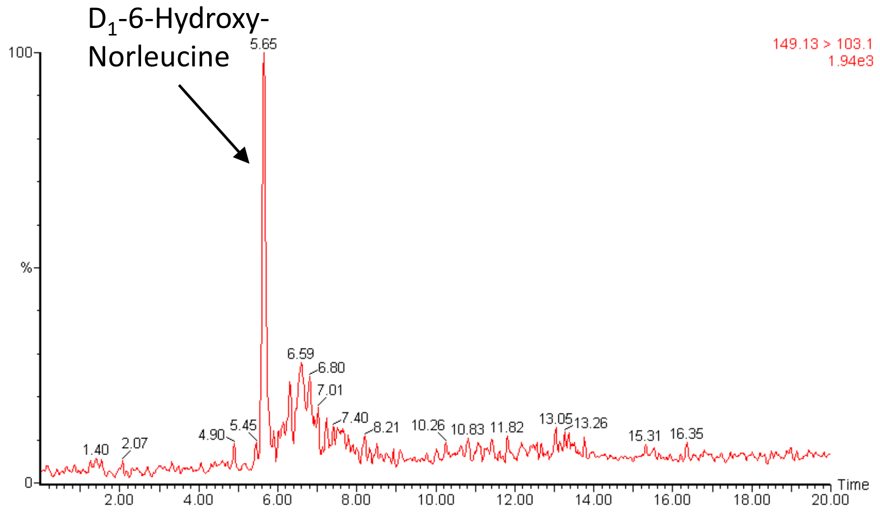

Above: m/z 149 trace showing the signal from $NaBD_4$ reduced unstretched tendon

Endogenous signal in unstretched tendon

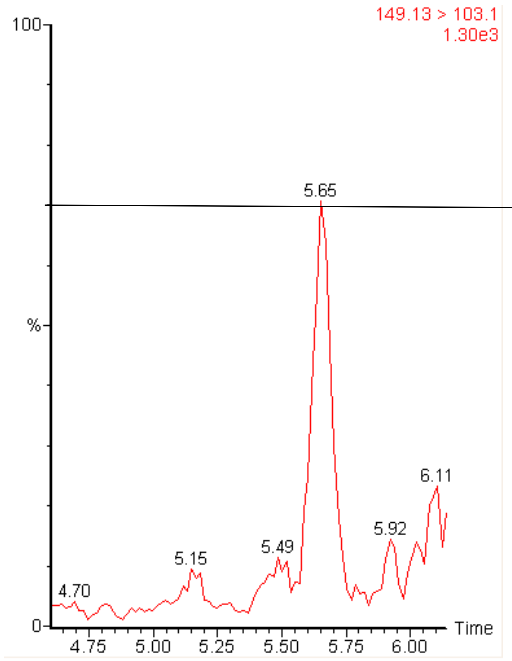

Endogenous signal in unstretched tendon plus $^{13}C_1$ isotope signal from standard spike

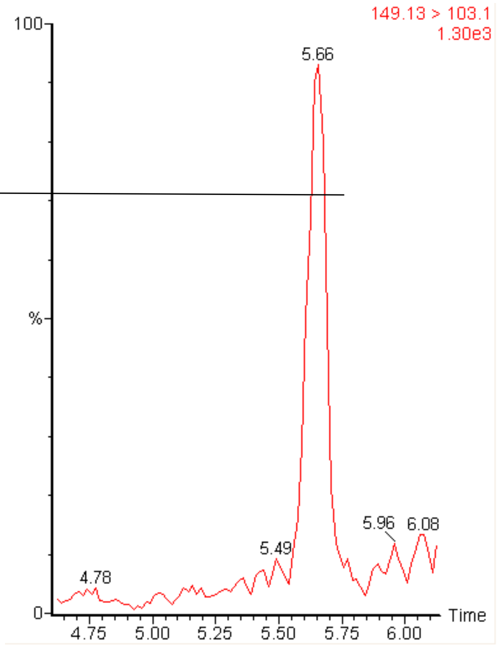

Allysine measurement		
**sample**	ng /mg tendon	mol/mol collagen*
**Pre-stretch control tendon**	89.27	0.179
**10% stretch 11 week tendon**	235.78	0.475

*The mol/mol collagen value is an approximation because it has been calculated by assuming that the tendon weight was 100% collagen. The result of this approximation is that the mol/mol collagen values shown here will be slightly underestimated. This has been done to allow comparison with the crosslink values in Figure 4. The tendon weight used was the dried and de-lipidated value.

Figure S3

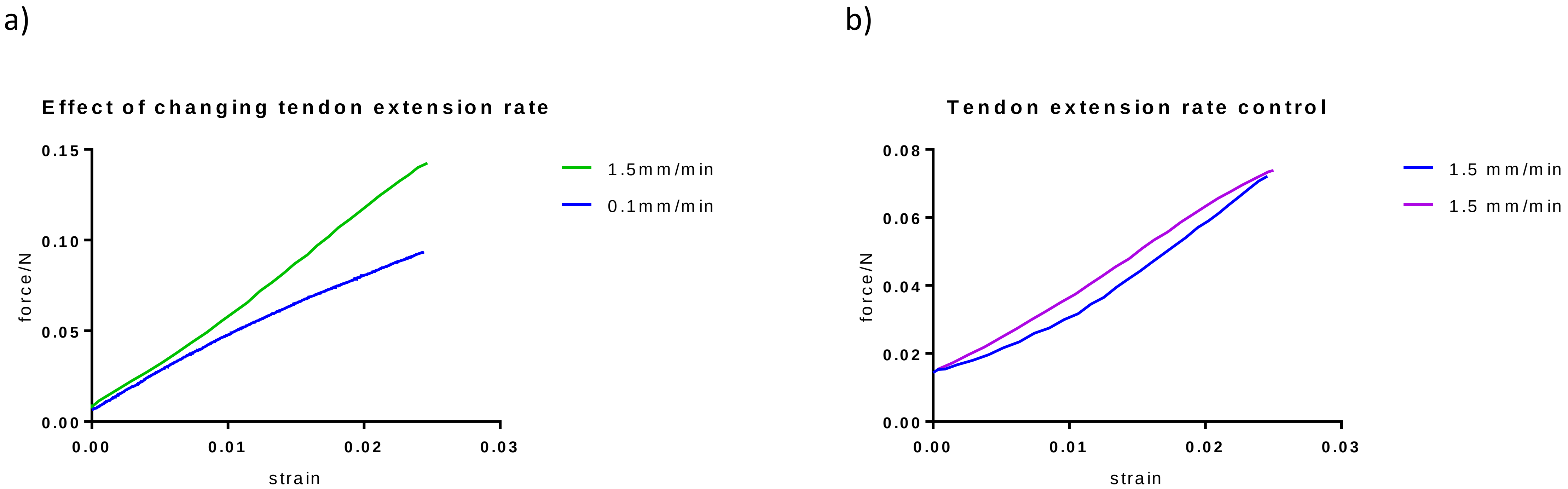

Figure S2 a) Example to show that the rate of increase in strain effects the resulting force and stiffness (slope). The pair are samples of the same tendon cut into two equal lengths. This is so that they are as similar as possible, the same tendon cannot be re-used because the act of stretching it changes its properties. b) Example control to show that two halves of a cut tendon behave similarly if extended at the same rate.
